# Risk Factors for Prolonged Length of Hospital Stay and Readmissions After Laparoscopic Sleeve Gastrectomy and Laparoscopic Roux-en-Y Gastric Bypass

**DOI:** 10.1007/s11695-017-2844-x

**Published:** 2017-07-31

**Authors:** Piotr Major, Michał Wysocki, Grzegorz Torbicz, Natalia Gajewska, Alicja Dudek, Piotr Małczak, Michał Pędziwiatr, Magdalena Pisarska, Dorota Radkowiak, Andrzej Budzyński

**Affiliations:** 1 2nd Department of General Surgery, Jagiellonian University Medical College, Kopernika 21 St., 31-501 Krakow, Poland; 2 Department of Endoscopic, Metabolic and Soft Tissue Tumors Surgery, Krakow, Poland; 3 Centre for Research, Training and Innovation in Surgery (CERTAIN Surgery), Krakow, Poland; 4 Students’ Scientific Group at 2nd Department of Surgery, JUMC, Krakow, Poland

**Keywords:** Bariatric surgery, Length of hospital stay, Risk factors, Readmission

## Abstract

**Background:**

Laparoscopic sleeve gastrectomy (LSG) and laparoscopic gastric bypass (LRYGB) are most commonly performed bariatric procedures. Laparoscopic approach and enhanced recovery after surgery (ERAS) protocols managed to decrease length of hospital and morbidity. However, there are patients in whom, despite adherence to the protocol, the length of stay (LOS) remains longer than targeted. This study aimed to assess potential risk factors for prolonged LOS and readmissions.

**Methods:**

The study was a prospective observation with a post-hoc analysis of bariatric patients in a tertiary referral university teaching hospital. Inclusion criteria were undergoing laparoscopic bariatric surgery. Exclusion criteria were occurrence of perioperative complications, prior bariatric procedures, and lack of necessary data. The primary endpoints were the evaluations of risk factors for prolonged LOS and readmissions.

**Results:**

Median LOS was 3 (2–4) days. LOS > 3 days occurred in 145 (29.47%) patients, 79 after LSG (25.82%) and 66 after LRYGB (35.48%; *p* = 0.008). Factors significantly prolonging LOS were low oral fluid intake, high intravenous volume of fluids administered on POD0, and every additional 50 km distance from habitual residence to bariatric center. The risk of hospital readmission rises with occurrence of intraoperative adverse events and low oral fluid intake on the day of surgery on.

**Conclusions:**

Risk factors for prolonged LOS are low oral fluid intake, high intravenous volume of fluids administered on POD0, and every additional 50 km distance from habitual residence. Risk factors for hospital readmission are intraoperative adverse events and low oral fluid intake on the day of surgery.

## Introduction

Nowadays, laparoscopic sleeve gastrectomy (LSG) and laparoscopic gastric bypass (LRYGB) are becoming first-line treatment for morbid obesity in Poland and worldwide, with widely accepted low risk for postoperative morbidity and low mortality [1–5]. In addition, treating patients in accordance with enhanced recovery after surgery (ERAS) protocols has reduced the length of stay (LOS) and decreased morbidity [6, 7]. Despite that, some patients with successful adherence to the protocol and uneventful peri- and post-operative period fail to meet discharge criteria and remain a longer period of time than targeted LOS. Shorter LOS may be associated with an increased rate of readmissions, and in some patients it results in readmission for variety of reasons [8–10].

The relationship between perioperative complications occurrence and prolonged LOS seems to be obvious, but the interesting question is what is affecting LOS in uncomplicated patients. Therefore, we analyzed risk factors for prolonged hospitalization and hospital readmission in order to predict patients without complications who might require longer hospital observation.

## Aim

Study aim was to identify risk factors for prolonged length of hospital stay and unplanned readmission after laparoscopic sleeve gastrectomy and laparoscopic Roux-en-Y gastric bypass among patients with uncomplicated postoperative course.

## Methods

The study was a prospective observation with a post-hoc analysis of patients who underwent bariatric surgery in a tertiary referral university teaching hospital. The indication for surgical treatment was taken from the IFSO-EC and EASO guidelines: body mass index (BMI) ≥ 35 kg/m^2^ with comorbidities or BMI of ≥ 40 kg/m^2^ [11, 12]. The inclusion criteria were written informed consent upon hospital admission acknowledging that the outcomes of perioperative treatment and follow-up may be analyzed retrospectively and used for research, age of 18 to 65 years, and eligibility for LSG or LRYGB. Exclusion criteria were occurrence of perioperative complications, prior bariatric procedures, and lack of necessary data. Study was designed according to STROBE Statement [13].

Patients were treated in accordance with the principles of multimodal ERAS pathway, including preoperative, intraoperative, and postoperative interventions [2, 6, 14–16]. Preoperative interventions included extensive perioperative counseling, shortened fluid fasts, preoperative high protein and carbohydrate drink, and optimized operating scheduling times. Intraoperatively, the optimized bariatric anesthetic protocol was introduced with the use of multimodal analgesia. There was no routine use of nasogastric tubes and intraabdominal drains. Postoperative interventions included early mobilization, analgesia without opioids, administration of IPP-antagonist, early enteral feeding, and discharge planning. Anti-thrombotic prophylaxis was administered up to 14 postoperative days with the use of Enoxaparine. Detailed information on protocol used in our clinic is presented below:

1. Preoperative counseling and patient’s education
2. Pre-operative carbohydrate loading (400 ml of Nutricia preOp® 2 h prior surgery)
3. Antithrombotic prophylaxis (Clexane® 40 mg sc. starting in the evening prior surgery)
4. Antibiotic prophylaxis (preoperative Ceftriaxone 2 g iv 30–60 min. prior surgery)
5. Laparoscopic surgery
6. Balanced intravenous fluid therapy (< 2500 ml intravenous fluids during the day of surgery, less than 150 mmol sodium). Indication for administration of i.v. fluids after surgery: vomiting, insufficient oral fluid intake (less than 500 ml before 7:00 pm, 7 h after surgery), insufficient diuresis (less than 500 ml of urine before 7:00 pm, 7 h after surgery). The target is to not use intravenous fluids in POD1. Fluids were given only if we observed absence of sufficient functional recovery: vomiting, insufficient oral fluid intake (less than 500 ml until 4:00 pm), insufficient diuresis, or biochemical symptoms of rhabdomyolysis
7. No nasogastric tubes postoperatively
8. No drains left routinely
9. TAP block and bariatric anesthesia protocol
10. Avoiding opioids, multimodal analgesia (oral when possible—Paracetamol 4 × 1 g, Ibuprofen 2 × 200 mg, Metamizole 2 × 500 mg, or Ketoprofen 2 × 100 mg); routine use of ondansetron, metoclopramide, and deksametazon at the end of the surgery
11. Postoperative oxygenation therapy (4–6 l/min)
12. Early oral feeding (oral nutritional supplement 4 h postoperatively—Nutrcia Nutridrink® or Nestlé Impact®, light hospital diet, and oral nutritional supplements on the first postoperative day, full hospital diet in the second postoperative day)
13. Early mobilization on the day of surgery (sitting on the bed 2 h after surgery, breathing exercises with physiotherapist, walking to the toilette, and walking along the corridor (at least 150 m) accompanied by an ERAS nurse or a family member. Full mobilization on the first postoperative day (getting out of bed, going to the toilette, walking along the corridor without support of the nurse, at least 4–6 h out of bed)

On admission, every patient was informed about the target length of stay of 3 days. Discharge criteria:

- Oral diet tolerance (solid food consumption, drinking at least 1500 ml of fluids)
- No need for i.v. drugs or fluids
- Balanced diuresis
- Pain of low magnitude, manageable with oral pain killers
- No fever
- Properly healing surgical wounds
- Physical activity at a level similar to pre-surgery time
- No complications requiring in-hospital observation
- Other’s people help available in the first days after surgery
- Possibility to return to hospital when suspecting complication development (means to contact, transportation from home)

Patients were scheduled for postoperative appointment 2 weeks after discharge, next 1 and 6 months after discharge. Surgical techniques for LSG and LRYGB were standardized in all cases to reduce study bias.

The primary endpoints were the evaluations of risk factors for prolonged length of stay and of risk factors for readmissions in the postoperative period in the group of patients without complications.

LOS was defined as the length of inpatient episode of care, calculated from the day of admission to the day of discharge, and based on the number of nights spent in hospital. The discharge occurred regardless of the day of the week. Patients were dichotomized into group of patients whose hospital stay was shorter or equal to the target LOS (≤3 days) and group of patients with prolonged LOS, defined as total length of stay in bariatric care unit longer than 3 days. Readmission was defined as hospitalization after discharge from bariatric care unit related to bariatric treatment within 30-days postoperative period.

Comparison of baseline patients data was done using Student’s *t* test or Mann-Whitney’s test for quantitative variables, while χ^2^ with or without Yates’ correction were used for qualitative variables. Univariate and multivariate logistic regression models were built to assess influence of patient- and treatment-related parameters on odds ratios (OR) with 95% confidence intervals (95% CI) for prolonged hospital stay and hospital readmissions. *P* value < 0.05 was considered statistically significant. Data were analyzed using Statistica version 12.0 PL (StatSoft Inc., Tulsa, Oklahoma, USA). Quantitative data are expressed as mean ± standard deviation or medians with interquartile range.

All procedures performed in studies involving human participants were in accordance with the ethical standards of the institutional and national research committee and with the 1964 Helsinki Declaration and its later amendments or comparable ethical standards. The study was approved by the Bioethics Committee of the Jagiellonian University.

## Material

**Fig. 1:**
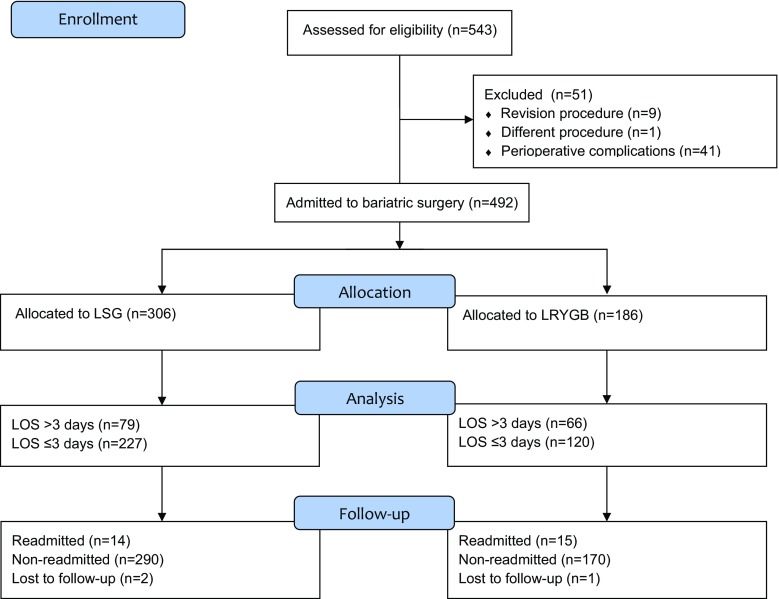
Study flowchart

From April 2009 to July 2016, 543 patients were treated for morbid obesity. Then, 492 patients met inclusion criteria and were submitted to laparoscopic sleeve gastrectomy or laparoscopic Roux-en-Y gastric bypass [310 females, 182 males, median age 42 (34–51) years] (Table 1). Flow chart of patients in the study is shown in Fig. 1.

**Table 1:** Patients and groups baseline characteristics

Parameter		All patients
		492 (100%)
Females, *n* (%)		310 (63%)
Males, *n* (%)		182 (37%)
Median age, years (IQR)		42 (34–51)
Median maximal preoperative BMI, kg/m^2^ (IQR)		46.56 (42.89–51.50)
Median BMI on a day of operation, kg/m^2^ (IQR)		45.09 (41.31–49.74)
∆BMI (IQR)		0.98 (0–2.42)
ASA class, *n* (%)	1	11 (2.30%)
	2	333 (69.52%)
	3	135 (28.18%)
Cardiovascular diseases, *n* (%)		87 (17.68%)
Hypertension, *n* (%)		335 (68.23%)
Prediabetes, *n* (%)		22 (4.47%)
Diabetes mellitus, *n* (%)		168 (34.29%)
Obstructive sleep apnea, *n* (%)		35 (7.11%)
Non-alcoholic fatty liver disease, *n* (%)		306 (62.45%)
Dyslipidemia, *n* (%)		278 (56.85%)

*IQR* interquartile range, *ASA class* American Society of Anesthesiologists physical status classification system

## Results

Median length of hospital stay (LOS) was 3 (2–4) days. LOS > 3 days occurred in 145 (29.47%) patients, 79 after LSG (25.82%), and 66 after LRYGB (35.48%; *p* = 0.008). Factors possibly prolonging length of hospital stay were analyzed in univariate logistic regression models. Maximal preoperative BMI > 51.50 kg/m^2^, LRYGB, operative time, intraoperative adverse events, high intraoperative volume of administered fluids, low postoperative oral fluid intake and diuresis, need for Furosemide forced diuresis, high postoperative intravenous fluid volume, postoperative tachycardia, occurrence of postoperative fever, postoperative nausea and vomiting, requirement for additional pain medication, every 50 km distance between place of residence and bariatric center were significantly related to the risk of prolonged LOS (LOS > 3 days vs. ≤ 3 days) (Table 2). Therefore, those possibly relevant factors were analyzed in multivariate logistic regression model (Table 3). Factors significantly prolonging LOS were low oral fluid intake, high intravenous volume of fluids administered on POD0, and every additional 50 km distance from habitual residence to bariatric center.

**Table 2:** Univariate logistic regression models for odds ratio of prolonged LOS (> 3 vs. ≤ 3 days)

Factors		OR	95% CI	*P*
Patient-related	Sex (M = 0, F = 1)	0.91	0.61–1.35	0.629
	Age (with every 1 year)	0.99	0.97–1.01	0.374
	Age (> 51 vs. ≤ 51 years)	1.07	0.68–1.70	0.761
	Maximal preoperative BMI (1 kg/m^2^)	1.03	0.99–1.06	0.056
	Maximal preoperative BMI (> 51.50 vs. ≤ 51.50 kg/m^2^)	1.55	1.01–2.39	*0*.*047*
	BMI on the day of operation (1 kg/m^2^)	1.02	0.99–1.06	0.119
	BMI on the day of operation (> 49.74 vs. ≤ 49.74 kg/m^2^)	1.41	0.91–2.18	0.124
Co-morbidities	ASA class (with every point)	1.36	0.91–2.04	0.133
	Cardio-vascular diseases (yes = 1, no = 0)	1.60	0.98–2.60	0.058
	Arterial hypertension (yes = 1, no = 0)	1.21	0.79–1.84	0.388
	Prediabetes (yes = 1, no = 0)	1.39	0.57–3.39	0.470
	Diabetes mellitus (yes = 1, no = 0)	1.25	0.84–1.88	0.271
	Obstructive sleep apnea (yes = 1, no = 0)	1.27	0.61–2.63	0.518
	Non-alcoholic fatty liver disease (yes = 1, no = 0)	0.94	0.63–1.40	0.752
	Dyslipidemia (yes = 1, no = 0)	1.36	0.91–2.02	0.131
	Malnutrition (yes = 1, no = 0)	1.42	0.92–2.19	0.113
Treatment-related	Type of procedure (LRYGB vs. LSG)	1.58	1.06–2.35	*0*.*023*
	Operative time (with every 1 min)	1.01	1.00–1.01	<*0*.*001*
	Operative time (> 150 vs. ≤ 150 min)	2.05	1.29–3.24	*0*.*002*
	Intraoperative adverse effects (yes = 1, no = 0)	3.99	1.51–10.53	*0*.*005*
	Intraoperative fluid volume administered (500 ml)	1.52	1.24–1.87	<*0*.*001*
Postoperative period	Oral fluid intake on POD0 (100 ml)	0.80	0.74–0.85	<*0*.*001*
	Oral fluid intake on POD1 (100 ml)	0.88	0.85–0.91	<*0*.*001*
	Oral fluid intake on POD2 (100 ml)	0.94	0.91–0.97	<*0*.*001*
	Oral fluid intake on POD3 (100 ml)	1.08	0.88–1.32	0.464
	Diuresis on POD0 (1000 ml)	1.85	1.44–2.40	<*0*.*001*
	Diuresis on POD1 (1000 ml)	1.20	0.91–1.57	0.190
	Diuresis on POD2 (1000 ml)	1.29	0.94–1.75	0.111
	Diuresis on POD3 (1000 ml)	28.95	0.08–10,888	0.261
	Furosemide forced diuresis (yes = 1, no = 0)	2.52	1.58–4.02	<*0*.*001*
	Intravenous fluid intake on POD0 (yes = 1, no = 0)	1.04	0.68–1.59	0.851
	Intravenous fluid volume on POD0 (500 ml)	1.55	1.04–2.31	*0*.*032*
	Intravenous fluid intake on POD1 (yes = 1, no = 0)	4.89	2.87–8.35	<*0*.*001*
	Intravenous fluid volume on POD1 (500 ml)	1.57	1.34–1.83	<*0*.*001*
	Intravenous fluid intake on POD2 (yes = 1, no = 0)	8.44	5.06–14.06	<*0*.*001*
	Intravenous fluid volume on POD2 (500 ml)	1.56	1.18–2.06	*0*.*002*
	Heart rate on POD0 (with every 1/min)	1.01	0.99–1.03	0.452
	Tachycardia on POD0 (yes = 1, no = 0)	9.82	1.08–89.19	*0*.*042*
	Heart rate on POD1 (with every 1/min)	0.99	0.97–1.01	0.420
	Tachycardia on POD1 (yes = 1, no = 0)	1.50	0.48–4.69	0.482
	Heart rate on POD2 (with every 1/min)	1.01	0.99–1.03	0.591
	Tachycardia on POD2 (yes = 1, no = 0)	2.04	0.50–8.32	0.319
	Heart rate on POD3 (with every 1/min)	1.00	0.97–1.03	0.940
	Tachycardia on POD3 (yes = 1, no = 0)	1.22	0.11–13.84	0.874
	CPK (> 1000 vs. < 1000 U/L)	1.55	0.87–2.74	0.135
	Postoperative fever (yes = 1, no = 0)	2.50	1.50–4.15	<*0*.*001*
	Postoperative nausea and vomiting (yes = 1, no = 0)	2.57	1.19–5.57	*0*.*016*
	Additional pain medication required (yes = 1, no = 0)	1.95	1.30–2.94	*0*.*001*
	Distance between the hospital and place of residence (50 km)	1.18	1.08–1.28	<*0*.*001*

Italic entries reached significance of *p* < 0.05 as stated in methodology

*ASA* class American Society of Anesthesiologists physical status classification system, *POD0* postoperative day 0, *POD1* postoperative day 1, *POD2* postoperative day 2, *POD3* postoperative day 3, *POD4* postoperative day 4, *CPK* creatine phosphokinase

**Table 3:** Multivariate logistic regression models for odds ratio of prolonged LOS (> 3 vs. ≤ 3 days)

	OR	95% CI	*P*
Maximal preoperative BMI (> 51.50 vs. ≤ 51.50 kg/m^2^)	1.11	0.39–3.13	0.849
Type of procedure (LRYGB vs. LSG)	0.74	0.25–2.21	0.590
Operative time (> 150 vs. ≤ 150 min)	1.11	0.36–3.38	0.859
Intraoperative adverse effects (yes = 1, no = 0)	1.88	0.37–9.49	0.439
Intraoperative fluid volume administered (500 ml)	0.75	0.45–1.24	0.255
Oral fluid intake on POD0 (100 ml)	0.77	0.67–0.88	<*0*.*001*
Intravenous fluid volume on POD0 (500 ml)	1.71	1.03–2.82	*0*.*036*
Diuresis on POD0 (1000 ml)	1.19	0.61–2.32	0.603
Furosemide forced diuresis (yes = 1, no = 0)	1.19	0.40–3.57	0.751
Tachycardia on POD0 (yes = 1, no = 0)	9.19	0.38–219.79	0.167
Postoperative fever (yes = 1, no = 0)	2.23	0.77–6.49	0.139
Postoperative nausea and vomiting (yes = 1, no = 0)	1.02	0.31–3.38	0.973
Additional pain medication required (yes = 1, no = 0)	0.88	0.36–2.15	0.775
Distance between the hospital and place of residence (50 km)	1.59	1.26–2.01	<*0*.*001*

Italic entries reached significance of *p* < 0.05 as stated in methodology

*POD0* postoperative day 0

Readmission rate in the study population was 5.89% (29 readmissions). Fourteen patients after LSG (4.58%) were readmitted, while 15 after LRYGB (8.06%; *p* = 0.172) (Table 4). Univariate logistic regression analyses revealed statistically significant impact of operative time, intraoperative adverse events, intraoperative volume of administered fluids, oral fluid intake, postoperative intravenous fluid administration, and prolonged LOS (> 3 days vs. ≤ 3 days) on the risk of hospital readmission (Table 5). Multivariate logistic regression model revealed significant impact of occurrence of intraoperative adverse events and low oral fluid intake on the day of surgery on the risk of hospital readmission (Table 6). Intraoperative adverse events which occurred in our unit are presented in Table 7.

**Table 4:** Reasons for readmission

	LSG	LRYGB
Operation site hernia	2 (0.65%)	3 (1.61%)
Cholecystitis	2 (0.65%)	3 (1.61%)
Linea alba herniation	0	2 (1.08%)
Internal hernia (Petersen space hernia)	0	1 (0.54%)
Perforated gastric ulcer	0	1 (0.54%)
GI bleeding	0	2 (1.08%)
Perianal abscess	0	1 (0.54%)
Pulmonary thrombosis	0	1 (0.54%)
Gastroesophageal reflux disease	3 (0.98%)	0
Fever of undetermined origin	1 (0.33%)	0
Chronic diarrhea	1 (0.33%)	0
Suspected ileus	1 (0.33%)	0
Dysphagia	4 (1.31%)	0
Lower abdomen pain	0	1 (0.54%)
Total	14/306 (4.58%)	15/186 (8.06%)

**Table 5:** Univariate logistic regression models of factors possibly affecting odds ratio of hospital readmission

Factors		OR	95% CI	*P*
Patient-related	Sex (M = 0, F = 1)	0.76	0.35–1.66	0.495
	Age (with every 1 year)	1.02	0.99–1.06	0.208
	Age (> 51 vs. ≤ 51 years)	1.14	0.47–2.77	0.765
	Maximal preoperative BMI (1 kg/m^2^)	1.05	0.99–1.10	0.094
	Maximal preoperative BMI (> 51.50 vs. ≤ 51.50 kg/m^2^)	1.44	0.63–3.28	0.382
	BMI on the day of operation (1 kg/m^2^)	1.03	0.97–1.09	0.404
	BMI on the day of operation (> 49.74 vs. ≤ 49.74 kg/m^2^)	1.44	0.63–3.28	0.382
Co-morbidities	ASA class (with every point)	1.54	0.69–3.43	0.287
	Cardio-vascular diseases (yes = 1, no = 0)	1.93	0.82–4.56	0.130
	Arterial hypertension (yes = 1, no = 0)	1.19	0.51–2.77	0.692
	Prediabetes (yes = 1, no = 0)	2.79	0.77–10.10	0.117
	Diabetes mellitus (yes = 1, no = 0)	0.91	0.40–2.06	0.823
	Obstructive sleep apnea (yes = 1, no = 0)	0.48	0.06–3.67	0.478
	Non-alcoholic fatty liver disease (yes = 1, no = 0)	0.93	0.42–2.02	0.847
	Dyslipidemia (yes = 1, no = 0)	1.65	0.73–3.74	0.227
	Malnutrition (yes = 1, no = 0)	1.57	0.66–3.74	0.304
Treatment-related	Type of procedure (LRYGB vs. LSG)	1.70	0.79–3.65	0.176
	Operative time (with every 1 min)	1.01	1.00–1.01	0.068
	Operative time (> 150 vs. ≤ 150 min)	2.31	1.02–5.24	*0*.*044*
	Intraoperative adverse effects (yes = 1, no = 0)	5.32	1.62–17.45	*0*.*006*
	Intraoperative fluid volume administered (500 ml)	1.78	1.27–2.50	*0*.*001*
Postoperative period	Oral fluid intake on POD0 (100 ml)	0.86	0.74–0.99	*0*.*030*
	Oral fluid intake on POD1 (100 ml)	0.94	0.88–1.01	0.072
	Oral fluid intake on POD2 (100 ml)	0.91	0.84–0.98	*0*.*010*
	Oral fluid intake on POD3 (100 ml)	0.92	0.81–1.04	0.162
	Diuresis on POD0 (1000 ml)	1.56	0.99–2.47	0.056
	Diuresis on POD1 (1000 ml)	0.74	0.40–1.36	0.337
	Diuresis on POD2 (1000 ml)	0.98	0.52–1.87	0.957
	Diuresis on POD3 (1000 ml)	2.01	0.93–4.34	0.072
	Furosemide-forced diuresis (yes = 1, no = 0)	0.78	0.33–1.87	0.577
	Intravenous fluid intake on POD0 (yes = 1, no = 0)	0.88	0.35–2.21	0.781
	Intravenous fluid volume on POD0 (500 ml)	0.05	0.004–0.68	*0*.*023*
	Intravenous fluid intake on POD1 (yes = 1, no = 0)	1.54	0.59–4.03	0.379
	Intravenous fluid volume on POD1 (500 ml)	1.19	0.96–1.47	0.113
	Intravenous fluid intake on POD2 (yes = 1, no = 0)	2.31	0.89–5.96	0.083
	Intravenous fluid volume on POD2 (500 ml)	0.95	0.63–1.42	0.791
	Heart rate on POD0 (with every 1/min)	1.01	0.96–1.05	0.767
	Tachycardia on POD0 (yes = 1, no = 0)	5.14	0.55–48.33	0.151
	Heart rate on POD1 (with every 1/min)	1.00	0.96–1.05	0.832
	Tachycardia on POD1 (yes = 1, no = 0)	1.67	0.21–13.57	0.628
	Heart rate on POD2 (with every 1/min)	1.01	0.97–1.05	0.709
	Tachycardia on POD2 (yes = 1, no = 0)	0.02	0.008–62.24	0.701
	Heart rate on POD3 (with every 1/min)	1.01	0.95–1.07	0.776
	Tachycardia on POD3 (yes = 1, no = 0)	6.94	0.57–84.03	0.126
	CPK (> 1000 vs. < 1000 U/L)	2.19	0.85–5.67	0.105
	Postoperative fever (yes = 1, no = 0)	1.15	0.38–3.52	0.804
	Postoperative nausea and vomiting (yes = 1, no = 0)	1.59	0.35–7.19	0.547
	Additional pain medication required (yes = 1, no = 0)	1.71	0.72–4.04	0.221
	Distance between the hospital and place of residence (50 km)	0.91	0.73–1.13	0.389
	Prolonged LOS (> 3 vs. ≤ 3 days)	2.57	1.19–5.56	*0*.*016*

Italic entries reached significance of *p* < 0.05 as stated in methodology

*ASA class* American Society of Anesthesiologists physical status classification system, *POD0* postoperative day 0, *POD1* postoperative day 1, *POD2* postoperative day 2, *POD3* postoperative day 3, *POD4* postoperative day 4, *LOS* length of stay, *CPK* creatine phosphokinase, *LOS* length of hospital stay

**Table 6:** Multivariate logistic regression model for hospital readmission

	OR	95% CI	*P*
Operative time (> 150 vs. ≤ 150 min)	2.79	0.34–22.94	0.335
Intraoperative adverse effects (yes = 1, no = 0)	4.20	1.17–151.14	*0*.*039*
Intraoperative fluid volume administered (500 ml)	1.88	0.72–4.92	0.192
Oral fluid intake on POD0 (100 ml)	0.54	0.29–0.99	*0*.*043*
Intravenous fluid volume on POD0 (500 ml)	0.03	0.001–1.18	0.059
Prolonged LOS (> 3 vs. ≤ 3 days)	0.31	0.03–3.42	0.332

Italic entries reached significance of *p* < 0.05 as stated in methodology

*POD0* postoperative day 0, *POD3* postoperative day 3, *LOS* length of stay

**Table 7:** Intraoperative adverse events

	All	Readmitted	Non-readmitted	*P*
Total	18/492 (3.66%)	4/29 (13.79%)	14/463 (3%)	*0*.*015*
Need for small intestine resection	6	2	4	*0*.*041*
Intraoperatively diagnosed staple line leakage	2	2	0	–
Inappropriate stapling that required modification of technique	2	0	2	–
Small intestine perforation	2	0	2	–
Excessive intraoperative bleeding	2	0	2	–
Intraoperatively diagnosed stricture	1	0	1	–
Intestinal serosa rupture	1	0	1	–
Probe immobilized with stapler	1	0	1	–
Intubation failure, cancelation of procedure	1	0	1	–

Italic entries reached significance of *p* < 0.05 as stated in methodology

## Discussion

We report outcomes of a study, where we sought to identify risk factors for prolonged length of hospital stay, despite instituted treatment according to modern model of perioperative care (ERAS). In our unit, our aim is to discharge the patient not as early as it is possible but as soon as patients reach full functional recovery. We demonstrated that with every 100 ml of oral fluid intake on the POD0, the risk for prolonged LOS decreased by 23%. As for i.v. fluids on POD0, the need for every 500 ml increased the risk for prolonged hospital stay 1.71 times. With every 50 km distance from the hospital to place of residence, the odds for prolonged LOS increased 1.59 times. We revealed that occurrence of intraoperative adverse events (after excluding patients with perioperative complications in study design) increased 4.2 times odds ratio for readmission. Additional data on which intraoperative events led to readmission are presented in Table 7. It should be emphasized that even though the result is statistically insignificant for particular types of events, the fact the readmissions occurred only after “Need for small intestine resection” or “Intraoperatively diagnosed staple line leakage” should alert the surgeon about possible readmission. High oral fluid intake on POD0 was protective factor decreasing risk for readmission 0.54 times.

Length of stay varies among bariatric centers due to differences in perioperative pathways and discharge criteria. Recent study on predictors for LOS, which included 9593 LRYGB, reported median length of stay of 2 days (range 0–544), in which 26% of patients required > 3 days of hospitalization [17]. In a study by Dallal et al., the hospital discharged 48% of bariatric patients by postoperative day (POD) 1, 85% by POD 2, and 96% by POD 3 [18]. Other studies on this matter usually reported LOS > 3 days as prolonged LOS, which is the same threshold as we had [19–21]. Different treatment protocols are administered nowadays for bariatric patients. Many researchers reported bariatric procedures performed in the fast-track surgery way; however, this requires additional actions to prepare and carry on treatment within 24-h hospital stay [22–24]. In our case, patients were admitted to the hospital in the afternoon prior to the operation day. Hospital stay was planned for 3 days and patients were obligated to follow criteria for discharge upon decision making. In summary, comparison between different bariatric centers could be troublesome, not only because of qualitative differences but also due to the applied approach to the treatment protocol.

The overall readmission rate in a study by Lois et al., which included 95,294 patients after bariatric surgery, was 5.7% [19]. Recent study by Sippey et al. reported an overall 30-day readmission rate after LSG or LRYGB of 5.1% [25]. Other authors reported readmission rates after those two procedures in the range of 1.87–14.16% [18, 26–33]. Despite many authors reporting more common readmissions after LRYGB compared with LSG [25, 27, 28, 34], one study documented higher rates after LSG [19]. In our study, overall readmission rate was 5.89%.

According to other studies, patient-related factors associated with prolonged LOS were age, higher preoperative BMI, male sex, number of preoperative comorbidities, and operative time [18, 35–38]. Regarding demographic factors in our study, they were not significantly related to the risk of prolonged LOS. Considering comorbidities, Carter et al. in a large study involving 500 hospitals in the National Surgical Quality Improvement Program (NSQIP) database showed that after gastric by-pass, longer hospitalization was predicted by diabetes, chronic obstructive pulmonary disease, hemorrhagic diathesis, chronic kidney disease, hypoalbuminemia, but with no other patient-related factors [17]. Sun et al. showed that patients with history of congestive heart failure, peripheral vascular, and kidney diseases were more prone to have longer LOS [39]. However, in our study, neither higher ASA class nor the presence of other comorbidities was related to the risk of prolonged LOS. We proved that in the end, LRYGB was not significantly related to prolonging LOS in comparison with LSG. Similar conclusions were found Weiner et al. [40]. We failed to determine what caused the lack of influence of patient’s health status or comorbidities on our endpoints. We hypothesize that it may be caused by individualized perioperative care, which was proven in another surgical disciplines [7, 41–43]. In studies by Reames et al., Carter et al., and Dallall et al., researchers concluded that the longer was the operative time, the greater was the risk for prolonged length of stay [17, 18, 20]. The same relationship was found in our study; however, a multivariate analysis revealed that it was contributing less to the risk of prolonged LOS. Nossaman et al. concluded that the volume of intravenous fluids administered during laparoscopic bariatric surgery significantly correlates to LOS and delays wound healing [37]. They showed that lower volumes of intraoperative fluid were significantly associated with longer LOS [37]. Contrary to that, we observed that patients who required larger amounts of intraoperative i.v. fluids were more prone to longer hospital stay; however, this correlation was not shown in the multivariate analysis. In our study, decreased amount of postoperative oral fluid intake (on POD0), need for i.v. fluids (POD0), and increasing distance between the hospital and place of residence were significantly contributing to increased risk of prolonged hospital stay.

In numerous papers, we found significant correlations of some patient-dependent factors and comorbidities on readmission rates, i.e., higher BMI and ASA class, diabetes mellitus, cardio-vascular, respiratory, renal co-morbidities, and chronic steroid intake, protein malnutrition were showed to be associated with increased risk of readmission [21, 25, 34, 35, 38, 39]. Treating patients in accordance to ERAS protocol probably contributed to non-significant relationship of preoperative ASA class and comorbidities with the risk of postoperative readmissions in our study [6, 7, 15, 16, 44]. We, as other researchers did, determined that BMI was not found to be a predictor of readmission [34, 45, 46].

In study by Sippey et al., the readmissions were higher after LRYGB compared with LSG [25]. In another, patients who underwent LRYGB had 60% higher risk for readmission in the 30-day postoperative time period compared to those who underwent LSG [34]. Contrary to this, we and Doumouras et al. did not observe similar relationship [26]. Operative time and intraoperative adverse events were also proved to increase risk of readmission [34, 47]. Postoperative complications were found to be associated with significant increase in readmissions in study by Khorgami et al. and other researchers [26, 34, 48–50]. In our study, multivariate analysis elicited independent influence of intraoperative adverse events and oral fluid intake on POD0 as factors associated with the risk of readmission. Regarding the fact that one of the most common reason for readmission after bariatric surgery is nausea/vomiting (12.95% in study by Aman et al. [28]), the decreased amount of oral fluid intake is a reasonable risk factor for readmission. We came to similar conclusions basing on our material.

Lois et al., Dallal et al., and Baker et al. concluded that in case of patients with LOS > 3 days, the risk of readmission was several times more greater than in on day hospitalizations [18, 19, 33]. Longer LOS was also a predictor of readmission in the study by Sun et al. [39]. In our study, patients with prolonged LOS (> 3 days) were 2.57 times more likely to be readmitted in comparison with patients whose LOS was ≤ 3 days; however, the result was only significant in the univariate analysis. Generally, it has to be emphasized that the factors we found to be increasing the risk of readmission are associated with first 24–48 after since the beginning of surgery. Early discharge is a result of how patients manages the treatment during the early hours. This way we are able to select patients who are at potentially greater risk of readmission.

## Limitation of the Study

The study did not focus on the potential differences arising from the severity of diabetes. Further study on the relationship between the severity of diabetes in bariatric patients and its impact on LOS and postoperative course is needed.

## Conclusion

Decreased oral fluid intake, need for increased i.v. fluid administration on the day of surgery, and longer distance from habitual residence to bariatric center are potential risk factors for prolonged hospital stay. Intraoperative adverse events increase risk for hospital readmission. Greater oral fluid intake on the day of procedure was associated with lower risk for readmission.
